# Assessment of early changes in ^3^H-fluorothymidine uptake after treatment with gefitinib in human tumor xenograft in comparison with Ki-67 and phospho-EGFR expression

**DOI:** 10.1186/1471-2407-13-525

**Published:** 2013-11-06

**Authors:** Songji Zhao, Yuji Kuge, Yan Zhao, Satoshi Takeuchi, Kenji Hirata, Toshiki Takei, Tohru Shiga, Hirotoshi Dosaka-Akita, Nagara Tamaki

**Affiliations:** 1 Department of Tracer Kinetics & Bioanalysis, Graduate School of Medicine, Hokkaido University, Sapporo, Japan; 2 Central Institute of Isotope Science, Hokkaido University, Sapporo, Japan; 3 Department of Medical Oncology, Hokkaido University, Sapporo, Japan; 4 Department of Nuclear Medicine, Graduate School of Medicine, Hokkaido University, Sapporo, Japan

**Keywords:** ^3^H-FLT, Gefitinib, Molecular-targeted therapy, A431, Athymic nude mice

## Abstract

**Background:**

The purpose of this study was to evaluate whether early changes in 3′-deoxy-3′-^3^H-fluorothymidine (^3^H-FLT) uptake can reflect the antiproliferative effect of gefitinib in a human tumor xenograft, in comparison with the histopathological markers, Ki-67 and phosphorylated EGFR (phospho-EGFR).

**Methods:**

An EGFR-dependent human tumor xenograft model (A431) was established in female BALB/c athymic mice, which were divided into three groups: one control group and two treatment groups. Mice in the treatment groups were orally administered a partial regression dose (100 mg/kg/day) or the maximum tolerated dose of gefitinib (200 mg/kg/day), once daily for 2 days. Mice in the control group were administered the vehicle (0.1% Tween 80). Tumor size was measured before and 3 days after the start of treatment. Biodistribution of ^3^H-FLT and ^18^F-FDG (%ID/g/kg) was examined 3 days after the start of the treatment. Tumor cell proliferative activity with Ki-67 was determined. Immunohistochemical staining of EGFR and measurement of phospho-EGFR were also performed.

**Results:**

High expression levels of EGFR and Ki-67 were observed in the A431 tumor. After the treatment with 100 and 200 mg/kg gefitinib, the uptake levels of ^3^H-FLT in the tumor were significantly reduced to 67% and 61% of the control value, respectively (0.39 ± 0.09, 0.36 ± 0.06, 0.59 ± 0.11%ID/g/kg for 100 mg/kg, 200 mg/kg, and control groups, respectively; *p* < 0.01 vs. control), but those of ^18^F-FDG were not. After the treatment with 100 and 200 mg/kg gefitinib, the expression levels of Ki-67 in the tumor were markedly decreased (4.6 ± 2.4%, 6.2 ± 1.8%, and 10.4 ± 5.7% for 100 mg/kg, 200 mg/kg, and control groups, respectively, *p* < 0.01 vs. control). The expression levels of the phospho-EGFR protein also significantly decreased (29% and 21% of the control value for 100, and 200 mg/kg, respectively *p* < 0.01 vs. control). There was no statistically significant difference in tumor size between pre- and post-treatments in each group.

**Conclusion:**

In our animal model, ^3^H-FLT uptake levels significantly decreased after the treatment with two different doses of gefitinib before a significant change in tumor size was observed. These results were confirmed by the immunohistochemical staining of Ki-67 and phospho-EGFR protein immunoassay. Thus, it was indicated that early changes in ^3^H-FLT uptake may reflect the antiproliferative effect of gefitinib in a mouse model of a human epidermoid cancer.

## Background

The epidermal growth factor receptor (EGFR) is a receptor tyrosine kinase that plays a crucial role in the signal transduction pathway, regulating key cellular functions such as proliferation, angiogenesis, metastasis, and evasion of apoptosis [1,2]. EGFR is highly overexpressed in numerous types of human cancers, including lung, stomach, and head and neck cancers, and is a strong prognostic factor [3-6].

Gefitinib, a selective small-molecule EGFR tyrosine kinase inhibitor, is widely used as a second- or third-line therapy for the treatment of patients with advanced non-small cell lung cancer (NSCLC) who failed to respond to standard chemotherapy [7]. Very recently, the European Medicine Agency has granted marketing authorization for gefitinib in patients with locally advanced or metastatic NSCLC with activating mutations of EGFR in all lines of therapy [8]. First-line gefitinib was approved in Korea for the treatment of patients with NSCLC who harbor the EGFR mutation [9]. However, gefitinib-induced interstitial lung disease (ILD) has been reported as a serious adverse effect [10,11], in addition to the common adverse effects of gefitinib including skin rash and diarrhea. To avoid the adverse effects and to effectively use the molecular targeted drug, it is necessary to accurately evaluate the tumor response early after the start of treatment. Such an evaluation method enables us to identify patients responsive to gefitinib and determine the treatment strategy: continuation or discontinuation of gefitinib therapy, or even a reduction in gefitinib dose. Indeed, re-administration at a reduced dose is a potential treatment strategy for patients who have once responded to, but later discontinued gefitinib treatment owing to severe adverse effects including ILD. The early and accurate assessment of treatment effects is particularly necessary in these patients. Recently, EGFR mutation, EGFR copy number, and EGFR protein expression are the three EGFR-related biomarkers that have been reported to be associated with the therapeutic benefit of gefitinib [12]. However, the therapeutic effect of gefitinib is not confined to patients whose tumors harbor EGFR mutation and other predictors of efficacy of this agent. In general, about 80% of NSCLCs with EGFR mutation respond to EGFR-TKIs, whereas 10% of tumors without EGFR mutations do so [13]. Although this observation provides highly valuable insights into the molecular mechanisms underlying sensitivity to EGFR-TKIs, none of the known clinical or molecular tumor characteristics allows the accurate prediction of tumor response at an early phase of treatment with gefitinib in an individual patient. Therefore, there is a clear need for new approaches to identify patients who will benefit from treatment with EGFR-TKIs. In this respect, imaging techniques that can be used to predict treatment outcome in an early phase of treatment are warranted.

X-ray computed tomography (CT) and magnetic resonance imaging (MRI) have commonly been used to evaluate the anti-tumor effect of cytotoxic and molecular targeted drugs by measuring tumor size. However, these anatomical imaging techniques have limited value because a relatively long time is required to obtain sufficient tumor size shrinkage with successful drug therapies. Thus, patients may have to endure adverse effects [14] and high medical costs [15] during the periods of desperate treatment. These limitations could be overcome using functional imaging techniques such as positron emission tomography (PET), because metabolic and physiologic changes in the tumor are likely to precede changes in size [16]. The quantitative nature of PET also contributes to the accurate determination of functional changes. In fact, PET imaging using 2-deoxy-2-^18^F-fluoro-D-glucose (^18^F-FDG) is increasingly used to assess early tumor response after chemotherapy [17]. On the other hand, the thymidine analog 3′-deoxy-3′-^18^F-fluorothymidine (^18^F-FLT) was also developed as a PET tracer for imaging tumor proliferation in vivo [18]. ^18^F-FLT uptake has been shown to reflect the activity of thymidine kinase-1 (TK1), an enzyme expressed during the DNA synthesis phase of the cell cycle. Owing to the phosphorylation of ^18^F-FLT by TK1, negatively charged ^18^F-FLT monophosphate is formed, resulting in intracellular trapping and accumulation of radioactivity [19]. Thus, this tracer is retained in proliferating cells through the activity of thymidine kinase. Accordingly, ^18^F-FLT PET could more appropriately evaluate the effects of signal transduction inhibitors whose main action mechanism is the inhibition of tumor cell proliferation, as compared with ^18^F-FDG PET [20]. Measurement of tumor proliferative activity by ^18^F-FLT PET may enable early and accurate assessment of the response to therapy with molecular targeted drugs [21].

Taken together, we aim to apply ^18^F-FLT PET for monitoring the antiproliferative effect of gefitinib. Several studies have shown that ^18^F-FLT PET is useful for the early evaluation of tumor response to anti-EGFR targeted therapy such as erlotinib and cetuximab [22-24]. However, there have been no studies on the usefulness of ^18^F-FLT PET for monitoring the antiproliferative effect of gefitinib, except for two reports [25,26]. Sohn et al. demonstrated that ^18^F-FLT PET can predict early responses to gefitinib treatment in patients with advanced pulmonary adenocarcinoma [25]. The effect of gefitinib on ^3^H-FLT uptake in vitro was studied previously by Su et al. [26]. Although several studies have indicated the ability of ^18^F-FLT or ^3^H-FLT to detect the effect of gefitinib [25,26], whether changes in ^18^F-FLT uptake can reflect the effect of gefitinib by comparing the level of ^18^F-FLT uptake with those of other proliferation or predictive markers, such as Ki-67 or phosphorylated EGFR, in an early phase of treatment has not been fully validated under a pathological condition.

Thus, in the present study, to determine whether early changes in ^3^H-FLT uptake can reflect the antiproliferative effect of gefitinib, we determined the changes in ^3^HFLT uptake level after the start of treatment at different doses of gefitinib in comparison with those in ^18^F-FDG uptake, Ki-67 expression, and phospho-EGFR levels in a human tumor xenograft (EGFR-dependent human tumor xenograft model, A431).

## Methods

### Radiopharmaceutical

[Methyl-^3^H (N)]-3′-fluoro 3′-deoxythymidine (^3^H-FLT) (specific activity, 74–370 GBq/mmol) was purchased from Moravek Biochemicals Inc. ^18^F-FDG was obtained from the Hokkaido University Hospital Cyclotron Facility, which produces the tracer for clinical use.

### Animal studies

All experimental protocols were approved by the Laboratory Animal Care and Use Committee of Hokkaido University. Nine-week-old female BALB/c athymic nude mice (supplied by Japan SLC, Inc., Hamamatsu, Japan) were used in all experiments. Room temperature was maintained between 23 and 25°C, and relative humidity was maintained between 45 and 60%. The institutional laboratory housing the cages provided a 12-hour light cycle and met all the criteria of the Association for Assessment and Accreditation of Laboratory Animal Care (AAALAC) International. The EGFR-dependent human tumor xenograft model was established in mice using the human epidermoid cancer cell line A431 (European Collection of Cell Cultures). A431 is a human cell line established from an epidermoid carcinoma of the vulva of an 85-year-old female patient, which has gene amplification and an unusually high number of EGF receptors [27]. A431 cells (5 × 10^6^ cells/0.1 ml) were inoculated subcutaneously into the right flank of the mice [28]. A431 xenograft is a recognized model for the testing of the biological effects on EGFR signaling [29]. When the tumors reached 5–8 mm in diameter, the mice were randomly divided into three groups, one control group (n = 8) and two treatment groups (n = 14). Mice in one treatment group were orally administered a partial regression gefitinib dose (100 mg/kg/day, n = 7) and those in the other treatment group the maximum tolerated gefitinib dose (200 mg/kg/day, n = 7), once daily for 2 days. Mice in the control group were given the vehicle (0.1% Tween 80). Gefitinib was purchased from Chugai Pharmaceutical Co., Ltd. (Tokyo, Japan). The doses of gefitinib have been widely used to evaluate its effects on human tumor xenografts [29-31]. Tumor size and body weight were measured before and 3 days after the start of treatment. Tumor volume was calculated using the formula: π/6 × larger diameter × (smaller diameter)^2^.

After overnight fasting, mice in the control and treatment groups were intravenously injected with a mixture of ^18^F-FDG (7.4 MBq) and ^3^H-FLT (0.185 MBq) 24 hours after the second treatment under light anesthesia. Sixty minutes after the injection, the mice were sacrificed, and tumor tissues and other organs were excised. Tumor tissues were cut into three pieces for radioactivity measurement, immunohistochemical staining and phospho-EGFR, respectively. The tissue and blood samples were weighed, and ^18^F-radioactivity was determined using a gamma-counter (1480 WIZARD 3"; Wallac Co., Ltd.). The samples were then solubilized with Soluene 350 (Packard Bioscience B.V.), and ^3^H-radioactivity was measured using a liquid scintillation counter (LSC-5100; Aloka Co., Ltd.) following the decay of ^18^F. Radioactivity uptake in the tissues was expressed as the percentage of injected dose per gram of tissue after being normalized to the animal’s weight (%ID/g) × kg. The tumor-to-muscle (T/M) ratios was calculated as (%ID/g) × kg. For the subsequent immunohistologic staining, tumor samples were formalin-fixed and paraffin-embedded. The remaining tumor samples were immediately frozen using liquid nitrogen for the subsequent phosphor-EGFR assay.

### Pathological studies

Formalin-fixed, paraffin-embedded, 3-μm-thick sections of tumor tissue were used for immunohistochemical staining. Immunohistochemical stainings of EGFR and Ki-67 (a tumor cell proliferation marker) was carried out using adjacent sections, in accordance with a standard procedure [32]. EGFR was stained using a monoclonal antibody (mAb) (mouse IgG1, Clone 31G7, Zymed, South San Francisco, CA) that recognizes the 170 kDa extracellular EGF binding domain. A mouse monoclonal antibody, clone MIB-1 (Dako, Carpinteria, CA) was used as a primary antibody for the staining of the nuclear antigen Ki-67. The Ki-67 labeling index was defined as the percentage of the number of positively stained cells with respect to the total number of cells in the entire field of the specimens.

### Phospho-EGFR assay

Phospho-EGFR (Tyr) was determined by a sandwich immunoassay method using a Bio-Plex phospho-EGFR (Tyr) assay kit (Bio-Plex phosphoprotein assay, Bio-Rad Laboratories, Inc.) in accordance with the manufacturer’s instructions. Briefly, the frozen tumor samples (3x3 mm) were homogenized in a lysing solution (Bio-Plex Cell Lysis Kit). The lysate was centrifuged to remove insoluble materials, and the aliquot (50 μl) was incubated with 50 μl of anti- phospho-EGFR (Tyr)-antibody-coupled beads in a 96-well plate for 18 hours at 20°C. After washing the beads, 50 μl of and EGFR specific biotinylated detection antibody was added and incubated with the beads for 30 minutes at room temperature. After washing three times streptavidin conjugated to a fluorescent protein, streptavidin-phycoerythrin (PE) (Bio-Plex phosphoprotein detection reagent kit, Bio-Rad Laboratories, Inc.) was added and incubated with the beads for 10 minutes at room temperature. Finally, after washing off unbound Streptavidin-PE, the beads were suspended in Bio-Rad assay buffer and analyzed on a Bio-Rad 96-well plate reader using the Bio-Plex 200 Array System (Bio-Rad Laboratories, Inc.). The median fluorescence intensity (MFI) was calculated from a standard curve using Bio-Plex Manager software (Bio-Rad Laboratories, Inc.) [33] and considered to be proportional to analyte concentration. The protein content in the lysates was determined by Bio-Rad DC protein assay (Bio-Rad Laboratories, Inc.).

### Statistical analyses

All values are expressed as means ± SD (standard deviation). One-way ANOVA was used to assess the significance of differences among the three groups. Bonferroni correction was implemented for post-hoc comparison. Paired Student’s *t*-test was performed to evaluate the significance of difference in tumor volume between pro- and post-treatments in each group. A value of *p* < 0.05 was considered significant. The statistical program Stat View 5.0 was used for data assessment.

## Results

### Studies of ^3^H-FLT and ^18^F-FDG biodistribution

Table 1 and 2 show the biodistribution and the tumor-to-muscle (T/M) ratios of ^3^H-FLT (Table 1) and ^18^F-FDG (Table 2). In the control group, the radioactivity derived from ^3^H-FLT was higher in the tumor than in other organs (Table 1). The ^18^F-FDG uptake level was higher in the tumor than in the blood and muscle, with relatively high ^18^F-FDG uptake levels in the heart, brown fat, and kidneys in the control mice (Table 2).

**Table 1:** **Biodistribution of **^**3**^**H-FLT in mice bearing A431 tumors**

**Tissue**	^**3**^**H-FLT uptake level ((%ID/g)×kg)**		
	**Control**	**Gefitinib 100**	**Gefitinib 200**
	**n = 8**	**n = 7**	**n = 7**
Blood	0.088 ± 0.009	0.080 ± 0.017	0.091 ± 0.012
Tumor	0.589 ± 0.112	0.393 ± 0.093*	0.360 ± 0.059*
Muscle	0.093 ± 0.008	0.088 ± 0.013	0.094 ± 0.008
Heart	0.094 ± 0.012	0.085 ± 0.012	0.092 ± 0.008
Brown fat	0.082 ± 0.010	0.076 ± 0.006	0.083 ± 0.009
Lung	0.091 ± 0.012	0.085 ± 0.011	0.094 ± 0.007
Brain	0.016 ± 0.001	0.015 ± 0.001	0.015 ± 0.001
Spleen	0.115 ± 0.026	0.113 ± 0.025	0.122 ± 0.033
Liver	0.103 ± 0.014	0.100 ± 0.017	0.105 ± 0.012
Kidney	0.147 ± 0.016	0.132 ± 0.020	0.138 ± 0.014
Skin	0.089 ± 0.019	0.094 ± 0.009	0.092 ± 0.010
Tumor/muscle ratio	6.3 ± 1.3	4.6 ± 1.4*	3.8 ± 0.6*

*p < 0.01 vs. Control.

**Table 2:** **Biodistribution of **^18^**F-FDG in mice bearing A431 tumors**

**Tissue**	^18^**F-FDG uptake level ((%ID/g)×kg)**		
	**Control**	**Gefitinib 100**	**Gefitinib 200**
	**n = 8**	**n = 7**	**n = 7**
Blood	0.046 ± 0.027	0.032 ± 0.019	0.037 ± 0.015
Tumor	0.129 ± 0.045	0.112 ± 0.023	0.111 ± 0.023
Muscle	0.022 ± 0.005	0.020 ± 0.007	0.020 ± 0.005
Heart	0.648 ± 0.587	0.709 ± 0.467	0.526 ± 0.587
Brown fat	0.372 ± 0.182	0.241 ± 0.090	0.290 ± 0.187
Lung	0.108 ± 0.017	0.100 ± 0.010	0.100 ± 0.010
Brain	0.171 ± 0.028	0.156 ± 0.020	0.172 ± 0.033
Spleen	0.115 ± 0.014	0.103 ± 0.013	0.113 ± 0.015
Liver	0.071 ± 0.033	0.055 ± 0.026	0.058 ± 0.018
Kidney	0.168 ± 0.029	0.122 ± 0.050	0.124 ± 0.036
Skin	0.162 ± 0.030	0.136 ± 0.042	0.149 ± 0.024
Tumor/muscle ratio	5.8 ± 1.4	5.9 ± 1.5	5.6 ± 1.1

Three days after the start of treatment with 100 and 200 mg/kg of gefitinib, the uptake levels of ^3^H-FLT in the tumor were significantly reduced to 67% and 61% of the control value, respectively. The T/M ratios of ^3^H-FLT uptake were also significantly decreased to 72% and 60% of the control value, respectively (Table 1). The uptake levels of ^18^F-FDG were not reduced significantly by the treatment (87% and 86% of the control value for 100 and 200 mg/kg gefitinib, respectively. The T/M ratios of ^18^F-FDG were not reduced significantly (102% and 97% of the control value for 100 and 200 mg/kg gefitinib, respectively) (Table 2). No significant differences were observed in mouse body weight among the three groups before and 3 days after the start of treatment. Mouse body weights were 19.6 ± 1.1 g for the control group and 18.8 ± 1.5 g and 19.0 ± 0.9 g for the 100 and 200 mg/kg gefitinib groups, and 18.6 ± 1.3 g for the control group and 17.3 ± 0.9 g and 17.7 ± 0.8 g for the 100 and 200 mg/kg gefitinib groups before and 3 days after the start of treatment, respectively.

### Pathological study

**Figure 1:**
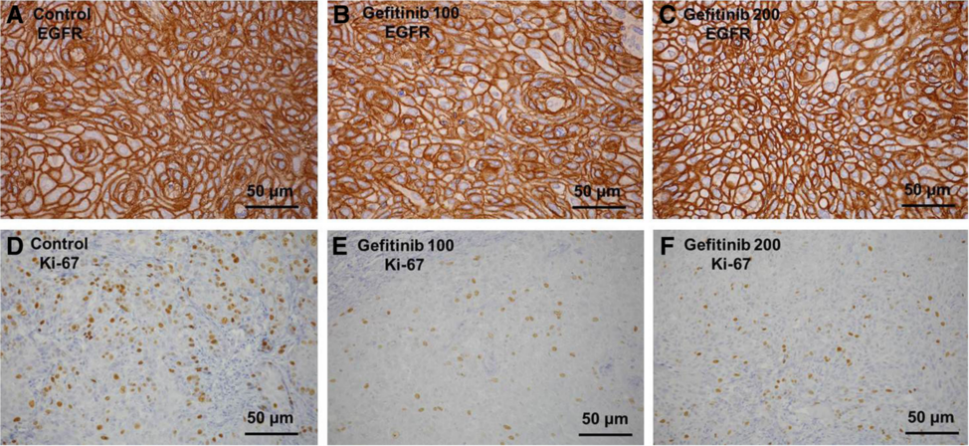
**Microscopy images of immunohistochemically stained EGFR (A-C) and Ki-67 (D-F) in tumor. ** Control, Gefitinib 100, and Gefitinib 200 indicate the control group, group treated with 100 mg/kg gefitinib, and group treated with 200 mg/kg gefitinib, respectively. Values given are mean ± SD.

Typical microscopy images of EGFR and Ki-67 immunostaining in the tumor are shown in Figure 1. A high expression level of EGFR was observed in the tumor cell membranes of control mice (Figure 1A). A high expression level of Ki-67 was also observed in the tumor cell nucleus of control mice (Figure 1D). After the treatment with 100 and 200 mg/kg gefitinib, the expression level of Ki-67 in the tumor markedly decreased (Figure 1E and F), whereas there was no significant change in the expression level of EGFR (Figure 1B and C).

**Figure 2:**
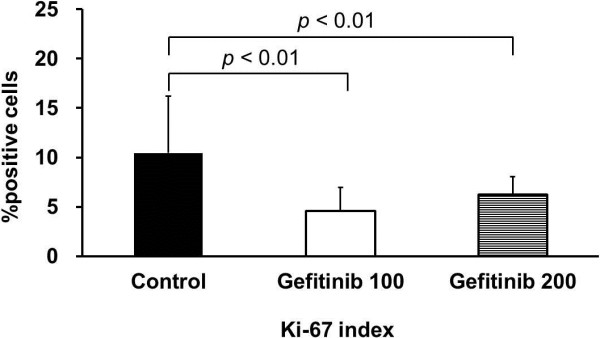
**Quantitative analysis of Ki-67 index in tumor.** Control, Gefitinib 100, and Gefitinib 200 indicate the control group, group treated with 100 mg/kg gefitinib, and group treated with 200 mg/kg gefitinib, respectively. Values given are mean ± SD.

**Figure 3:**
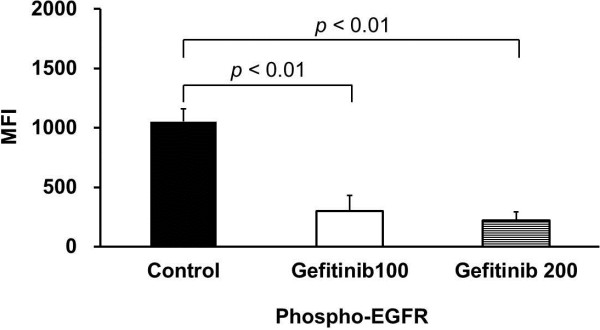
**Quantitative analysis of phospho-EGFR protein expression level in tumor.** Control, Gefitinib 100, and Gefitinib 200 indicate the control group, group treated with 100 mg/kg gefitinib, and group treated with 200 mg/kg gefitinib, respectively. Values given are mean ± SD.

**Figure 4:**
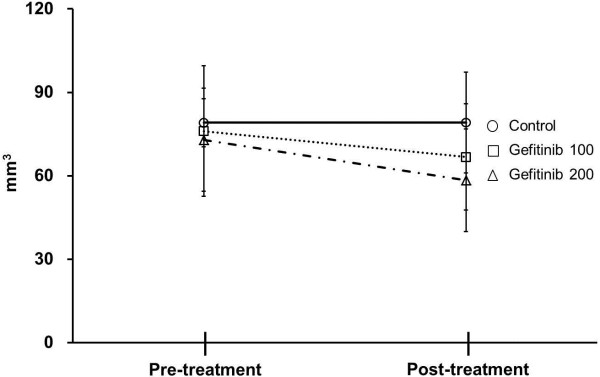
**Comparison of tumor size between pre- and post-treatments in each group.** Control, Gefitinib 100, and Gefitinib 200 indicate the control group, group treated with 100 mg/kg gefitinib, and group treated with 200 mg/kg gefitinib, respectively. Pre- and post-treatment indicate before and 3 days after the start of treatment with gefitinib; Values given are mean ± SD.

Results of quantitative analysis of Ki-67 positive cells (index) in the tumor are summarized in Figure 2. As compared with the control, the Ki-67 index in the tumor tissues was significantly decreased after the gefitinib treatment (Ki-67 index: 4.6 ± 2.4% for 100 mg/kg; 6.2 ± 1.8% for 200 mg/kg; 10.4 ± 5.7% for control group; *p* < 0.01 for both treated groups vs. control group). Results of quantitative analysis of phospho-EGFR (Tyr) protein in the tumor are summarized in Figure 3. The median flurorescence intensity (MFI) of the phospho-EGFR (Tyr) protein in the tumor also significantly decreased after gefitinib treatment: 301.1 ± 131.4 MFI for 100 mg/kg (29% of control); 220.0 ± 70.8 MFI for 200 mg/kg (21% of control); 1052.0 ± 106.2 MFI for control group; *p* < 0.01 for both treated groups vs. control group. There was no statistically significant difference in tumor size between before and 3 days after the treatment in each group (Figure 4).

## Discussion

After the treatment with two different doses of gefitinib, the ^3^H-FLT uptake levels in the tumor were significantly decreased at an early time point (Table 1). These early changes in tumor proliferation activity were confirmed by our pathological studies that including immunohistochemical staining of the Ki-67 (Figure 2) and phospho-EGFR assay (Figure 3). There was no statistically significant difference in tumor size between pre- and post-treatments in each group. Thus, the measurement of tumor proliferative activity using ^3^H-FLT may enable early accurate assessmentof the response to therapy with a molecular targeted drug, gefitinib, in human tumor xenografts.

Kawano et al. reported that the phospho-EGFR expression level significantly correlates with the response to gefitinib treatment [34]. They showed that a high level of basal EGFR activation (phospho-EGFR) is an important indicator of sensitivity to gefitinib. When ligands bind to the receptor, the molecule is phosphorylated (phospho-EGFR) by constitutive tyrosine kinases, activating downstream pathways [35]. Gefitinib blocks EGFR tyrosine kinases and prevents epidermal growth factor-induced proliferation of cultured cells. It inhibits growth and causes regression in human tumor xenografts overexpressing EGFR [29]. In our study, these effects of gefitinib were confirmed by the phospho-EGFR assay and analysis of ^3^H-FLT uptake in the tumor. Namely, phosphor-EGFR expression level was markedly decreased after the gefitinib treatment, which was accompanied by the reduction in ^3^H-FLT uptake level. Shen, et al. [36] also reported that the expression level of phospho-EGFR in lung cancer cells treated with gefitinib for 2 days was lower than that in non-treated cells. Su et al. [26] reported that the growth inhibitory effect of gefitinib was parallel to the inhibition of EFGR phosphorylation in a gefitinib-sensitive cell line (NSCLC H3255). These data strongly support our results in confirming the proof of the mechanism of the EGFR inhibitor gefitinib. Thus, our findings suggest that ^3^H-FLT can reflect EGFR activation and can be a predictor of the tumor response to gefitinib in human tumor xenograft.

Several clinical trials have demonstrated that ^18^F-FLT can be used for imaging a various tumor types and that there is strong correlation between ^18^F-FLT uptake level and proliferation index (Ki-67) in individual tumors [37-40]. Although TK1 is not a specific proliferation marker, TK1 is regulated within the cell cycle [41], and the ^18^F-FLT uptake level within tumors usually reflects the fraction of tumor cells in the S-phase, in which the TK1 expression level is the highest. The TK1 activity is high in proliferating cells and low in dormant cells. In our study, the antiproliferative effect of gefitinib was confirmed by the Ki-67 and ^3^H-FLT uptake in the tumor. Namely, the expression level of Ki-67 was markedly decreased after the gefitinib treatment, which was accompanied by a reduction in ^3^H-FLT uptake level.

Because ^18^F-FLT PET findings reflect the proliferation of tumor cells, this method is more suitable for detecting the early therapeutic effect than conventional modalities such as CT and MRI, which are based on sequential measurements of tumor size. Recently, several investigators used ^18^F-FLT PET to evaluate treatment responses in animal models or humans following molecular targeted therapy [22,42]. However, the potentials of ^18^F-FLT PET for monitoring the antiproliferative effect of gefitinib have not been clarified. Our present findings suggest that ^3^H-FLT can predict the therapeutic effect of gefitinib at a very early time point (2-days after the start of gefitinib treatment) during which changes in tumor size cannot be detected yet. Su et al. [26] also showed that a marked decrease (~ 90%) in ^3^H-FLT uptake in NSCLC H3255 cells was observed 2 days after exposure to two different doses of gefitinib. The in vitro data supported our results in confirming the proof of the mechanism of the EGFR inhibitor gefitinib. Because molecular targeted drugs are used for patients with advanced stage cancer, it is very important to determine their therapeutic effects as early as possible. If the therapeutic effects can be predicted at a very early time point, it will be possible to select the clinically optimal treatment and reduce medical costs in advance. In the present study, ^3^H-FLT uptake level significantly decreased in a dose-dependent manner after the treatment with gefitinib. If the therapeutic effects can be predicted quantitatively and dose-dependently, ^18^F-FLT PET can also be applied to evaluate the therapeutic effect of gefitinib re-administration with dose reduction in patients who have once responded to but later discontinued this treatment owing to severe adverse events including ILD. As the precise management of a gefitinib responder having severe adverse events remains to be established, ^18^F-FLT PET may provide a potential means for the management of gefitinib responders having severe adverse events.

It is very important to compare the level of ^3^H-FLT uptake with that of ^18^F-FDG uptake, as ^18^F-FDG is the most widely used tracer for tumor imaging by PET. In our study, however, the ^18^F-FDG uptake level in the tumor was not significantly reduced by the treatment with gefitinib. In addition, the ^18^F-FDG uptake level in the tumor was lower than those in most of the other organs including the heart, brown fat, and kidneys. Mamede et al. reported that the level of ^18^F-FDG uptake by the tumors in immunodeficient (athymic nu/nu) mice was significantly lower than that in immunocompetent mice [43]. Therefore, our mouse model, BALB/c athymic nude mice bearing human epidermoid cancer (A431), dose not seem to be suitable for evaluating the potentials of ^18^F-FDG. Further studies, including comparisons between ^18^F-FLT and ^18^F-FDG uptake levels in mouse or rat allograft tumor models and in patients, are necessary to compare the potentials of ^18^F-FLT PET and ^18^F-FDG PET and to demonstrate the advantages of ^18^F-FLT PET for the early and accurate detection of the antiproliferative effect of gefitinib.

It seems better to measure the mice tumors in 3 dimensions (3.14/6 × (length × width × depth)) rather than using the smallest diameter for both the width and the depth (3.14/6 × longest diameter × (smallest diameter)^2^). Thus, to measure accurate tumor volumes, in vivo studies are necessary. Other limitations of our study were that ^3^H-FLT was used instead of ^18^F-FLT and only one tumor model (A431) was used to compare the uptake of ^3^H-fluorothymidine with the uptake of ^18^F-FDG, Ki67 and phospho-EGFR after the treatment with two different doses of gefitinib. ^18^F-FLT and other tumor models should be used to confirm our present results.

## Conclusions

In our animal model, the ^3^H-FLT uptake level significantly decreased after the treatment with two different doses of gefitinib before a significant change in tumor size was observed. These findings were confirmed by the immunohistochemical staining of Ki-67 and phospho-EGFR assay. Thus, it was indicated that early changes in ^3^H-FLT uptake may reflect the antiproliferative effect of gefitinib in a mouse model of human epidermoid cancer. ^18^F-FLT PET could be applied for early clarification of the therapeutic effect of gefitinib for selecting the clinically optimum treatment strategy and minimizing the fatal adverse effects.

## Competing interests

The authors declare that they have no competing interests.

## Authors’ contributions

SZ designed the experiments, drafted the manuscript and performed the data analysis, immunohistochemical staining of Ki-67, and phospho-EGFR protein immunoassay. YK conceived of the study, performed data analysis and revised the manuscript. YZ and ST helped in the study design, and also acquired and analyzed data. KH, TT, TS, and DH participated in discussion and data interpretation. NT conceived of the study and revised the manuscript. All authors read and approved the final manuscript.

## Pre-publication history

The pre-publication history for this paper can be accessed here:

http://www.biomedcentral.com/1471-2407/13/525/prepub

## References

[B1] Carmeliet P Mechanisms of angiogenesis and arteriogenesis Nat Med 2000 6 389 395 10742145 10.1038/74651

[B2] Wieduwilt MJ Moasser MM The epidermal growth factor receptor family: biology driving targeted therapeutics Cell Mol Life Sci 2008 65 1566 1584 18259690 10.1007/s00018-008-7440-8 PMC3060045

[B3] Normanno N De Luca A Bianco C Strizzi L Mancino M Maiello MR Carotenuto A De Feo G Caponigro F Salomon DS Epidermal growth factor receptor (EGFR) signaling in cancer Gene 2006 366 2 16 16377102 10.1016/j.gene.2005.10.018

[B4] Hirsch FR Varella-Garcia M Bunn PA Jr Di Maria MV Veve R Bremmes RM Barón AE Zeng C Franklin WA Epidermal growth factor receptor in non-small-cell lung carcinomas: correlation between gene copy number and protein expression and impact on prognosis J Clin Oncol 2003 21 3798 3807 12953099 10.1200/JCO.2003.11.069

[B5] Galizia G Lieto E Orditura M Castellano P Mura AL Imperatore V Pinto M Zamboli A De Vita F Ferraraccio F Epidermal growth factor receptor (EGFR) expression is associated with a worse prognosis in gastric cancer patients undergoing curative surgery World J Surg 2007 31 1458 1468 17516110 10.1007/s00268-007-9016-4

[B6] Chung CH Ely K McGavran L Varella-Garcia M Parker J Parker N Jarrett C Carter J Murphy BA Netterville J Burkey BB Sinard R Cmelak A Levy S Yarbrough WG Slebos RJ Hirsch FR Increased epidermal growth factor receptor gene copy number is associated with poor prognosis in head and neck squamous cell carcinomas J Clin Oncol 2006 24 4170 4176 16943533 10.1200/JCO.2006.07.2587

[B7] Kris MG Natale RB Herbst RS Lynch TJ Jr Prager D Belani CP Schiller JH Kelly K Spiridonidis H Sandler A Albain KS Cella D Wolf MK Averbuch SD Ochs JJ Kay AC Efficacy of gefitinib, an inhibitor of the epidermal growth factor receptor tyrosine kinase, in symptomatic patients with non-small cell lung cancer: a randomized trial JAMA 2003 290 2149 2158 14570950 10.1001/jama.290.16.2149

[B8] De Luca A Normanno N Predictive biomarkers to tyrosine kinase inhibitors for the epidermal growth factor receptor in non-small-cell lung cancer Curr Drug Targets 2010 11 851 864 20388064 10.2174/138945010791320773

[B9] Sun JM Lee KH Kim SW Lee DH Min YJ Yun HJ Kim HK Song HS Kim YH Kim BS Hwang IG Lee K Jo SJ Lee JW Ahn JS Park K Ahn MJ Gefitinib versus pemetrexed as second-line treatment in patients with nonsmall cell lung cancer previously treated with platinum-based chemotherapy (KCSG-LU08-01): an open-label, phase 3 trial Cancer 2012 118 6234 6242 22674612 10.1002/cncr.27630

[B10] Inoue A Saijo Y Maemondo M Gomi K Tokue Y Kimura Y Ebina M Kikuchi T Moriya T Nukiwa T Severe acute interstitial pneumonia and gefitinib Lancet 2003 361 137 139 12531582 10.1016/S0140-6736(03)12190-3

[B11] Ando M Okamoto I Yamamoto N Takeda K Tamura K Seto T Ariyoshi Y Fukuoka M Predictive factors for interstitial lung disease, antitumor response, and survival in non-small-cell lung cancer patients treated with gefitinib J Clin Oncol 2006 24 2549 2556 16735708 10.1200/JCO.2005.04.9866

[B12] Fukuoka M Wu YL Thongprasert S Sunpaweravong P Leong SS Sriuranpong V Chao TY Nakagawa K Chu DT Saijo N Duffield EL Rukazenkov Y Speake G Jiang H Armour AA To KF Yang JC Mok TS Biomarker analyses and final overall survival results from a phase III, randomized, open-label, first-line study of gefitinib versus carboplatin/paclitaxel in clinically selected patients with advanced non-small-cell lung cancer in Asia (IPASS) J Clin Oncol 2011 29 2866 2874 21670455 10.1200/JCO.2010.33.4235

[B13] Mitsudomi T Kosaka T Yatabe Y Biological and clinical implications of EGFR mutations in lung cancer Int J Clin Oncol 2006 11 190 198 16850125 10.1007/s10147-006-0583-4

[B14] Lynch TJ Jr Kim ES Eaby B Garey J West DP Lacouture ME Epidermal growth factor receptor inhibitor-associated cutaneous toxicities: an evolving paradigm in clinical management Oncologist 2007 12 610 621 17522250 10.1634/theoncologist.12-5-610

[B15] Mittmann N Au HJ Tu D O’Callaghan CJ Isogai PK Karapetis CS Zalcberg JR Evans WK Moore MJ Siddiqui J Prospective cost-effectiveness analysis of cetuximab in metastatic colorectal cancer: evaluation of National Cancer Institute of Canada Clinical Trials Group CO.17 trial J Natl Cancer Inst 2009 101 1182 1192 19666851 10.1093/jnci/djp232

[B16] Vansteenkiste J Fischer BM Dooms C Mortensen J Positron-emission tomography in prognostic and therapeutic assessment of lung cancer: systematic review Lancet Oncol 2004 5 531 540 15337482 10.1016/S1470-2045(04)01564-5

[B17] Takahashi R Hirata H Tachibana I Shimosegawa E Inoue A Nagatomo I Takeda Y Kida H Goya S Kijima T Yoshida M Kumagai T Kumanogoh A Okumura M Hatazawa J Kawase I Early [18F]fluorodeoxyglucose positron emission tomography at two days of gefitinib treatment predicts clinical outcome in patients with adenocarcinoma of the lung Clin Cancer Res 2012 18 220 228 22019513 10.1158/1078-0432.CCR-11-0868

[B18] Shields AF Grierson JR Dohmen BM Machulla HJ Stayanoff JC Lawhorn-Crews JM Obradovich JE Muzik O Mangner TJ Imaging proliferation in vivo with [F-18]FLT and positron emission tomography Nat Med 1998 4 1334 1336 9809561 10.1038/3337

[B19] Toyohara J Waki A Takamatsu S Yonekura Y Magata Y Fujibayashi Y Basis of FLT as a cell proliferation marker: comparative uptake studies with [3H]thymidine and [3H]arabinothymidine, and cell-analysis in 22 asynchronously growing tumor cell lines Nucl Med Biol 2002 29 281 287 11929696 10.1016/s0969-8051(02)00286-x

[B20] Waldherr C Mellinghoff IK Tran C Halpern BS Rozengurt N Safaei A Weber WA Stout D Satyamurthy N Barrio J Phelps ME Silverman DH Sawyers CL Czernin J Monitoring antiproliferative responses to kinase inhibitor therapy in mice with 3′-deoxy-3′-18F-fluorothymidine PET J Nucl Med 2005 46 114 120 15632041

[B21] Yang DJ Kim EE Inoue T Targeted molecular imaging in oncology Ann Nucl Med 2006 20 1 11 16485568 10.1007/BF02985584

[B22] Ullrich RT Zander T Neumaier B Koker M Shimamura T Waerzeggers Y Borgman CL Tawadros S Li H Sos ML Backes H Shapiro GI Wolf J Jacobs AH Thomas RK Winkeler A Early detection of erlotinib treatment response in NSCLC by 3′-deoxy-3′-[F]-fluoro-L-thymidine ([F]FLT) positron emission tomography (PET) PLoS One 2008 3 e3908 19079597 10.1371/journal.pone.0003908 PMC2592703

[B23] Takeuchi S Zhao S Kuge Y Zhao Y Nishijima K Hatano T Shimizu Y Kinoshita I Tamaki N Dosaka-Akita H 18F-fluorothymidine PET/CT as an early predictor of tumor response to treatment with cetuximab in human lung cancer xenografts Oncol Rep 2011 26 725 730 21667030 10.3892/or.2011.1338

[B24] Atkinson DM Clarke MJ Mladek AC Carlson BL Trump DP Jacobson MS Kemp BJ Lowe VJ Sarkaria JN Using fluorodeoxythymidine to monitor anti-EGFR inhibitor therapy in squamous cell carcinoma xenografts Head Neck 2008 30 790 799 18286491 10.1002/hed.20770 PMC3942889

[B25] Sohn HJ Yang YJ Ryu JS Oh SJ Im KC Moon DH Lee DH Suh C Lee JS Kim SW ^18^F]Fluorothymidine positron emission tomography before and 7 days after gefitinib treatment predicts response in patients with advanced adenocarcinoma of the lung Clin Cancer Res 2008 14 7423 7429 19010859 10.1158/1078-0432.CCR-08-0312

[B26] Su H Bodenstein C Dumont RA Seimbille Y Dubinett S Phelps ME Herschman H Czernin J Weber W Monitoring tumor glucose utilization by positron emission tomography for the prediction of treatment response to epidermal growth factor receptor kinase inhibitors Clin Cancer Res 2006 12 5659 5667 17020967 10.1158/1078-0432.CCR-06-0368

[B27] Merlino GT Xu YH Ishii S Clark AJ Semba K Toyoshima K Yamamoto T Pastan I Amplification and enhanced expression of the epidermal growth factor receptor gene in A431 human carcinoma cells Science 1984 224 417 419 6200934 10.1126/science.6200934

[B28] Akizawa H Zhao S Takahashi M Nishijima K Kuge Y Tamaki N Seki K Ohkura K In vitro and in vivo evaluations of a radioiodinated thymidine phosphorylase inhibitor as a tumor diagnostic agent for angiogenic enzyme imaging Nucl Med Biol 2010 37 427 432 20447553 10.1016/j.nucmedbio.2010.01.005

[B29] Wakeling AE Guy SP Woodburn JR Ashton SE Curry BJ Barker AJ Gibson KH ZD1839 (Iressa): An Orally active inhibitor of epidermal growth factor signaling with potential for cancer therapy Cancer Res 2002 62 5749 5754 12384534

[B30] Matar P Rojo F Cassia R Moreno-Bueno G Di Cosimo S Tabernero J Guzmán M Rodriguez S Arribas J Palacios J Baselga J Combined epidermal growth factor receptor targeting with the tyrosine kinase inhibitor gefitinib (ZD1839) and the monoclonal antibody cetuximab (IMC-C225): superiority over single-agent receptor targeting Clin Cancer Res 2004 10 6487 6501 15475436 10.1158/1078-0432.CCR-04-0870

[B31] Hara F Aoe M Doihara H Taira N Shien T Takahashi H Yoshitomi S Tsukuda K Toyooka S Ohta T Shimizu N Antitumor effect of gefitinib ('Iressa’) on esophageal squamous cell carcinoma cell lines in vitro and in vivo Cancer Lett 2005 226 37 47 16004931 10.1016/j.canlet.2004.12.025

[B32] Zhao S Kuge Y Tsukamoto E Mochizuki T Kato T Hikosaka K Hosokawa M Kohanawa M Tamaki N Effects of insulin and glucose loading on FDG uptake in experimental malignant tumours and inflammatory lesions Eur J Nucl Med 2001 28 730 735 11440033 10.1007/s002590100517

[B33] Griffin SM Chen IM Fout GS Wade TJ Egorov AI Development of a multiplex microsphere immunoassay for the quantitation of salivary antibody responses to selected waterborne pathogens J Immunol Methods 2011 364 83 93 21093445 10.1016/j.jim.2010.11.005

[B34] Kawano D Yano T Shoji F Ito K Morodomi Y Haro A Miura N Takenaka T Yoshino I Maehara Y The influence of intracellular epidermal growth factor receptor (EGFR) signal activation on the outcome of EGFR tyrosine kinase inhibitor treatment for pulmonary adenocarcinoma Surg Today 2011 41 818 823 21626329 10.1007/s00595-011-4514-2

[B35] Arteaga CL The epidermal growth factor receptor: from mutant oncogene in nonhuman cancers to therapeutic target in human neoplasia J Clin Oncol 2001 19 32S 40S 11560969

[B36] Shen L Li Z Shen S Niu X Yu Y Li Z Liao M Chen Z Lu S The synergistic effect of EGFR tyrosine kinase inhibitor gefitinib in combination with aromatase inhibitor anastrozole in non-small cell lung cancer cell lines Lung Cancer 2012 78 193 200 22985911 10.1016/j.lungcan.2012.08.012

[B37] Vesselle H Grierson J Muzi M Pugsley JM Schmidt RA Rabinowitz P Peterson LM Vallières E Wood DE In vivo validation of 3′deoxy-3′-[(18)F]fluorothymidine ([(18)F]FLT) as a proliferation imaging tracer in humans: correlation of [(18)F]FLT uptake by positron emission tomography with Ki-67 immunohistochemistry and flow cytometry in human lung tumors Clin Cancer Res 2002 8 3315 3323 12429617

[B38] Buck AK Schirrmeister H Hetzel M Von Der Heide M Halter G Glatting G Mattfeldt T Liewald F Reske SN Neumaier B 3-deoxy-3-[(18)F]fluorothymidine-positron emission tomography for noninvasive assessment of proliferation in pulmonary nodules Cancer Res 2002 62 3331 33314 12067968

[B39] Francis DL Freeman A Visvikis D Costa DC Luthra SK Novelli M Taylor I Ell PJ In vivo imaging of cellular proliferation in colorectal cancer using positron emission tomography Gut 2003 52 1602 1606 14570730 10.1136/gut.52.11.1602 PMC1773856

[B40] Wagner M Seitz U Buck A Neumaier B Schultheiss S Bangerter M Bommer M Leithäuser F Wawra E Munzert G Reske SN 3′-[18F]fluoro-3′-deoxythymidine ([18F]-FLT) as positron emission tomography tracer for imaging proliferation in a murine B-Cell lymphoma model and in the human disease Cancer Res 2003 63 2681 2687 12750297

[B41] Wang N He Q Skog S Eriksson S Tribukait B Investigation on cell proliferation with a new antibody against thymidine kinase 1 Anal Cell Pathol 2001 23 11 19 11790855 10.1155/2001/658312 PMC4618205

[B42] Herrmann K Wieder HA Buck AK Schöffel M Krause BJ Fend F Schuster T Meyer zum Büschenfelde C Wester HJ Duyster J Peschel C Schwaiger M Dechow T Early response assessment using 3′-deoxy-3′-[18F]fluorothymidine-positron emission tomography in high-grade non-Hodgkin’s lymphoma Clin Cancer Res 2007 13 3552 3558 17575218 10.1158/1078-0432.CCR-06-3025

[B43] Mamede M Saga T Ishimori T Nakamoto Y Sato N Higashi T Mukai T Kobayashi H Konishi J Differential uptake of (18)F-fluorodeoxyglucose by experimental tumors xenografted into immunocompetent and immunodeficient mice and the effect of immunomodification Neoplasia 2003 5 179 183 12659691 10.1016/s1476-5586(03)80010-6 PMC1502404

